# Diversity of dermal fibroblasts as major determinant of variability in cell reprogramming

**DOI:** 10.1111/jcmm.14316

**Published:** 2019-04-13

**Authors:** Anna Maria Sacco, Immacolata Belviso, Veronica Romano, Antonia Carfora, Fabrizio Schonauer, Daria Nurzynska, Stefania Montagnani, Franca Di Meglio, Clotilde Castaldo

**Affiliations:** ^1^ Department of Public Health, School of Medicine University of Naples Federico II Naples Italy

**Keywords:** cell reprogramming, fibroblast, induced pluripotent stem cells (iPSCs), mesenchymal stem cells (MSCs)

## Abstract

Induced pluripotent stem cells (iPSCs) are adult somatic cells genetically reprogrammed to an embryonic stem cell‐like state. Notwithstanding their autologous origin and their potential to differentiate towards cells of all three germ layers, iPSC reprogramming is still affected by low efficiency. As dermal fibroblast is the most used human cell for reprogramming, we hypothesize that the variability in reprogramming is, at least partially, because of the skin fibroblasts used. Human dermal fibroblasts harvested from five different anatomical sites (neck, breast, arm, abdomen and thigh) were cultured and their morphology, proliferation, apoptotic rate, ability to migrate, expression of mesenchymal or epithelial markers, differentiation potential and production of growth factors were evaluated in vitro. Additionally, gene expression analysis was performed by real‐time PCR including genes typically expressed by mesenchymal cells. Finally, fibroblasts isolated from different anatomic sites were reprogrammed to iPSCs by integration‐free method. Intriguingly, while the morphology of fibroblasts derived from different anatomic sites differed only slightly, other features, known to affect cell reprogramming, varied greatly and in accordance with anatomic site of origin. Accordingly, difference also emerged in fibroblasts readiness to respond to reprogramming and ability to form colonies. Therefore, as fibroblasts derived from different anatomic sites preserve positional memory, it is of great importance to accurately evaluate and select dermal fibroblast population prior to induce reprogramming.

## 1 INTRODUCTION

Induced pluripotent stem cells (iPSCs) are adult somatic cells genetically reprogrammed to an embryonic stem cell (ESC)‐like state. Since pioneering works that led to the successful reprogramming of mouse^1^ and human^2^, ^3^ fibroblasts, iPSCs have been obtained from somatic cells of several other species.^4^, ^5^, ^6^

Remarkable similarity of iPSCs to ESC, along with their origin from adult somatic cells, make iPSCs a tremendously valuable tool for regenerative medicine, disease modelling, drug discovery and testing,^7^, ^8^, ^9^ while avoiding the ethical concerns associated with ESC.

Notwithstanding iPSCs functional resemblance to ESC, their clinical application is still prevented by severe technical problems, mostly related to both reprogramming technology and low efficiency of reprogramming. Although reprogramming technology has been significantly improved by integration‐free methods based on episomal vectors,^10^ synthetic modified mRNA^11^ or direct delivery of reprogramming proteins,^12^ efficiency of reprogramming of human cells is still as low as 2% with integration‐free methods, and 6.2% at best with integration methods.^13^ Several enhancers and barriers of reprogramming have been described thus far. Accordingly, novel strategies to activate enhancers or inhibit barriers are emerging.^14^ Recently, reprogramming efficiencies of 80%‐100% were achieved by genetic combinatorial modulation of specific signalling pathways.^15^ These results shed light on the mechanisms governing cell reprogramming, but do not allow transcending the limitations to iPSC clinical translation. Additionally, evidence supporting variability in efficiency of reprogramming and in properties of iPSCs in accordance with the cell type from which iPSCs were generated was also reported.^16^, ^17^ Adult dermal fibroblast has been the first human cell successfully reprogrammed to iPSCs,^3^ and, to date, it is still the most used human cell for reprogramming. Even though the potential of other adult somatic cells has been examined, fibroblasts are still the most suitable cell source to generate iPSC. Intriguingly, peripheral blood cells (PBCs) and urine‐derived cells (HUCs) were emerging as alternate candidates for easiness of harvesting, but the very low efficiency of PBCs reprogramming and the very high inter‐individual variation in HUCs number excreted with urine,^13^ strengthened fibroblast role in human iPSCs production. Undoubtedly, dermal fibroblasts are easily accessible and propagated in culture with a single skin punch biopsy. However, according to gene expression profile analysis independently performed by different groups comparing fibroblasts from different anatomic sites, embryonic spatial organisation of fibroblast differentiation and positional memory are partially retained in adult fibroblast.^18^, ^19^ Furthermore, recently reported phenotypic and functional diversity of dermal fibroblasts^20^ might need to be considered when planning reprogramming of dermal fibroblasts to iPSCs.

On this basis, we hypothesize that dermal fibroblasts differ not only in their gene expression profile, but also in other biological characteristics that might be, even partially, responsible for different response to reprogramming technology. To test our hypothesis we compared the morphology, expression of specific markers, production of soluble factors, proneness to apoptosis, proliferation rate, ability to migrate and differentiation potential of adult human dermal fibroblasts isolated from different anatomic sites and analysed any difference occurring among cell populations.

## 2 MATERIALS AND METHODS

### 2.1 Tissue samples

Skin fragments from five different anatomic sites (n = 25, five necks, five breasts, five arms, five abdomens and five thighs) of patients (n = 25, mean age 41.04 ± 7.624, all female patients) undergoing plastic surgery were harvested. Patients provided written, informed consent and specimens were collected, without patient identifiers, following protocols approved by the University Hospital Federico II and in conformity with the principles outlined in the Declaration of Helsinki.

### 2.2 Cell culture

Samples were minced and fragments of about 2 × 1 mm, length by width, were placed under sterile coverglasses in 35 mm culture plates and cultured in DMEM (Sigma‐Aldrich, St. Louis, MO) with 10% FBS (Fetal Bovine Serum) (Sigma‐Aldrich) and 0.5% Penicillin‐Streptomycin (Sigma‐Aldrich), at 37°C in 5% CO_2_. Plates were checked daily at an inverted phase‐contrast microscope (Olympus, Tokyo, Japan), and medium was replaced every 3 days. Outgrowth of cells was documented by digital image acquisition (Olympus). Confluent fibroblasts from all regions were synchronised by being placed in 0.1% serum for 48 hours before being trypsinized and plated in the presence of 10% serum, as previously described.^21^ In order to avoid any effect because of the native environment, all primary fibroblasts were cultured under the same condition in vitro for five passages.^18^ Then, passage 5 fibroblasts from different anatomic sites were cultured for 1 week to evaluate their features and behaviour in vitro. All experiments were performed in triplicate.

### 2.3 Scratch wound assay

1.6 × 10^5^ cells/35 mm dish were plated to create a confluent monolayer. Dishes were cultured for approximately 48 hours at 37°C to allow cells to adhere and spread. Cell monolayer was scratched in a straight line with a p10 sterile pipette tip. Fresh medium was pipetted in the dish, after one wash with medium to remove debris. Culture plates were then placed under a phase‐contrast microscope (Nikon, Tokyo, Japan) equipped with stage incubator (Okolab, Pozzuoli, Italy), and the migration of fibroblasts at both edges of the wound was documented acquiring one picture every 10 minutes for 12 hours by digital camera (Nikon). Data were analysed by NIS Elements software (Nikon) and expressed as mean speed of migration ± SE.

### 2.4 Immunocytochemistry and proliferation index

7.5 × 10^4^ cells/35 mm dish were plated and cultured for 4 days in DMEM with 10% FBS. Cells were then fixed in 4% paraformaldehyde and immunostained. After three brief washes in PBS, cells were blocked with 10% donkey serum (Sigma‐Aldrich), then incubated for 1 hour at 37°C in humidified chamber with primary antibodies targeting vimentin, cadherin, smooth muscle actin, Factor VIII (all from Sigma‐Aldrich), CD90, (Cluster of Differentiation 90) CD105 (both from Abcam, Cambridge, UK) or Ki67 (Leica Biosystems, Wetzlar, Germany). After washing, cells were incubated with matching secondary antibody (Jackson ImmunoResearch, Newmarket, UK) either fluorescein or rhodamine‐conjugated. After a further wash, stained area of culture dish was mounted with coverglasses in Vectashield mounting medium with DAPI (4',6‐Diamidino‐2‐Phenylindole) (Vector Laboratories, Burlingame, CA). Observation, evaluation and documentation were performed by fluorescence microscope (Nikon). For ki67 staining, three independent observers counted all cells counterstained with DAPI and all ki67‐positive cells in the stained area. Results were averaged and proliferation index was calculated and expressed as mean percentage of cycling cells ± SE.

### 2.5 Apoptotic index

To determine apoptotic index of fibroblast from all regions, 7.5 × 10^4^ cells/35 mm culture dish were plated and cultured for 3 days, then fixed in 1% paraformaldehyde. Apoptotic cells were detected in situ by the indirect TUNEL (Terminal deoxynucleotidyl transferase dUTP nick end labeling) method using the ApopTag Fluorescein In Situ Apoptosis Detection Kit (Merck Millipore, Darmstadt, Germany), following supplied protocol. Then, three independent observers counted all cells counterstained with DAPI and all apoptotic cells in the stained area, under a fluorescence microscope (Nikon). Numbers were averaged and apoptotic index was calculated and expressed as mean percentage of apoptotic cells ± SE.

### 2.6 Gene expression profile analysis

Culture dishes(5 × 10^5^ cells/60 mm) were plated and cultured for 7 days, then processed for real‐time PCR analysis as previously described.^22^ Total RNA was extracted in Isol‐RNA Lysis Reagent (5Prime, Hamburg, Germany), dissolved in RNase‐free water and its final concentration determined at the NanoDrop 1000 spectrophotometer (Thermo Scientific, Waltham, MA). RNA from each sample was reverse transcribed into cDNA with QuantiTect Reverse Trascription Kit (Qiagen, Hilden, Germany) and gene expression was quantified by real‐time qPCR using PrecisionPLUS qPCR Master Mix (Primer Design, Southampton, UK). The primer assays for genes typical for mesenchymal cells are included in Table 1. All samples were tested in triplicate with the housekeeping gene (GAPDH, Glyceraldehyde‐3‐Phosphate Dehydrogenase) to correct for variations in RNA quality and quantity. Melt curve analysis was performed to assess uniformity of product formation, primer dimer formation and amplification of non‐specific products. Comparative quantification of target genes expression in the samples was performed based on cycle threshold (Ct) and using the ΔΔCt method. Numbers were averaged and expressed as mean ± SE.

**Table 1:** Primers of genes analysed by real‐time PCR

Gene symbol	Forward sequence	Reverse sequence	Amplicon length (nt)
GAPDH	5′‐CTCTCTGCTCCTCCTGTTCG‐3′	5′‐ACGACCAAATCCGTTGACTC‐3′	114
CD90	5′‐CTAGTGGACCAGAGCCTTCG‐3′	5′‐GCCCTCACACTTGACCAGTT‐3′	96
CD105	5′‐CCTCTACCTCAGCCCACACT‐3′	5′‐CTGTCTAACTGGAGCAGGAACTC‐3′	92
CD146	5′‐AGCCAAACATCCAGGTCAAC‐3′	5′‐TACCCGTTCCTCCCTACACA‐3′	88
ECM2	5′‐ATCCTTTTCAAGTTTTCCTGGAG‐3′	5′‐TGCCCTTTACCAAACAGTGTC‐3′	80
FN1	5′‐ACCGAGGTGACTGAGACCAC‐3′	5′‐GACACAACGATGCTTCCTGA‐3′	137
WISP1	5′‐TGGCAGCAGTGACAGCA‐3′	5′‐GGAGCTGGGGTAAAGTCCAT‐3′	88

### 2.7 Growth factor array

Dermal fibroblasts from all five regions (neck, breast, arm, abdomen and thigh) were plated at medium density (8 × 10^4^ cells/35 mm) and cultured in DMEM with 10% FBS. When cells reached confluence, serum‐free FibroGRO medium (Millipore, Burlington, MA) was used instead of DMEM for the next 3 days. Culture medium was then collected from fibroblasts of all dermal regions and assayed in the Human Growth Factor Array C1 (Raybiotech, Norcross, GA) to simultaneously detect 41 targets. The procedure was performed in strict accordance with manufacturer's directions. Briefly, array membranes were blocked with blocking buffer for 30 minutes at room temperature, and then 1 mL of culture medium was added to each membrane and incubated at room temperature for 2.5 hours. Membranes were then washed three times in wash buffer I and twice in wash buffer II, then incubated with biotin‐conjugated antibody overnight at 4°C. After further washes, membranes were incubated for 2 hours at room temperature with horseradish peroxidase (HRP)‐conjugated streptavidin and washed one last time to remove unbound reagents. All incubation steps were performed with agitation on orbital shaker. Membranes were then developed with the detection buffer, exposed to film and processed by autoradiography. Numerical comparison of the signal densities of growth factors known to influence or modulate reprogramming was performed as previously described.^23^ Briefly, spot signal densities from the scanned images of arrays were obtained using ImageJ densitometry software (https://imagej.nih.gov/ij/download.html). The background was then subtracted from the densitometry data, and the obtained values were normalised to the positive control signals. Data were expressed as the mean ± SE.

### 2.8 Differentiation potential

Passage 5 fibroblasts were seeded onto 35 mm standard plastic culture dishes (BD Falcon) in adipogenic, chondrogenic and osteogenic induction media as previously described.^24^ Dishes were checked daily at an inverted phase‐contrast microscope (Olympus) and morphological changes occurring were documented. The culture medium was replaced every 3 days and fibroblasts were kept in culture for 21 days. Then, cells were rinsed in PBS and fixed for 20 minutes in 4% neutral buffered paraformaldehyde. Differentiation was confirmed by histological staining using specific staining kits (Bio‐optica, Milan, Italy) and performed in accordance with manufacturer's protocols. Adipogenic differentiation was analysed using Oil red O staining as an indicator of intracellular lipid accumulation, chondrogenic differentiation was analysed using Alcian blue staining as an indicator of sulphated glucose‐aminoglycan‐rich extracellular matrix while osteogenic differentiation was analysed using von Kossa staining as an indicator of calcified extracellular matrix.

### 2.9 Reprogramming

Dermal fibroblasts from each anatomic site at passage 5 (n = 4) were reprogrammed using the Stemgent StemRNA‐NM Reprogramming Kit (Reprocell, Glasgow, UK), according to the manufacturer's protocol. Briefly, on day 0, a total of 2.5 × 10^4^ cells was seeded on each well of a 24‐well plate coated with hESC‐qualified Matrigel (Corning, Corning, NY) and cultured overnight at 37°C in 5% CO_2, _in Fibroblast Expansion Medium, prepared from Advanced‐DMEM (ThermoFisher Scientific), supplemented with 10% FBS (Sigma‐Aldrich) and Glutamax Supplement (ThermoFisher Scientific). On day 1, medium was changed to the iPSC cultivation medium NutriStem Medium (Reprocell) and transfection was performed after 6 hours using NM‐RNA Reprogramming Transfection Complex obtained by mixing the NM‐RNA Reprogramming Cocktail diluted in Opti‐MEM Reduced Serum Medium (reagent A) and Lipofectamine RNAiMAX Transfection Reagent, diluted in Opti‐MEM Reduced Serum Medium as well (reagent B) (all by ThermoFisher Scientific), according to the manufacturer's indication. Cells were then incubated for 15‐18 hours. Medium was, then, replaced and cells were allowed to recover for 6 hours. After the recovery time, cells were transfected and incubated again. Overnight 15‐18 hour transfections followed by 6 hour recovery times were repeated for a total of four times, as recommended by manufacturer. Starting from day 5, cells were kept in culture in NutriStem Medium, changing the medium on a daily basis. The formation of colonies was monitored everyday and documented using a phase‐contrast microscope (Nikon) equipped with a stage incubator (Okolab). Three independent observers counted the emerging colonies at day 7 and 14 after the last transfection at the phase‐contrast microscope (Nikon), while the size of colonies was measured by the NIS Elements software (Nikon). Numbers were then averaged and expressed as mean ± SE.

### 2.10 Statistical analysis

Data were analysed by GraphPad Prism 5.0 (GraphPad Software, La Jolla, CA), using one‐way ANOVA test and Tukey's post test. A value of *P* ≤ 0.05 identified any statistically significant difference.

## 3 RESULTS

### 3.1 *Fibroblast morphology *in vitro

**Figure 1:**
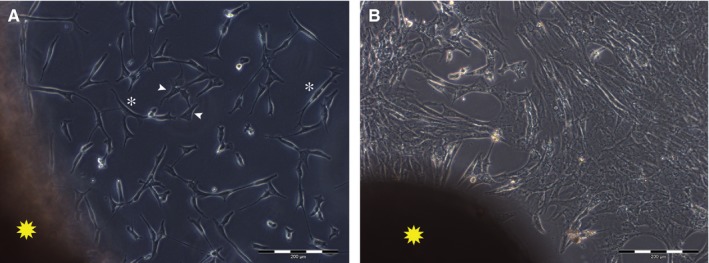
Representative images of fibroblasts in vitro. A, outgrowth from the skin fragment (yellow star) of spindle‐shaped (white asterisks) and star‐shaped (white arrowheads) fibroblasts in culture. B, fibroblasts in culture reached confluence in a time ranging between 14 and 21 d. Scale bar length is 200 µm

Outgrowth of fibroblasts occurred after 6‐7 days of culture (Figure 1A). Cells adhered to plastic culture dishes, where they spread and, in a time ranging from 14 to 21 days, they reached confluence (Figure 1B). Although most of the cells had spindle‐shaped morphology, star‐shaped fibroblasts were observed in all culture dishes (Figure 1A).

### 3.2 Proliferation and apoptotic indices, ability to migrate

**Figure 2:**
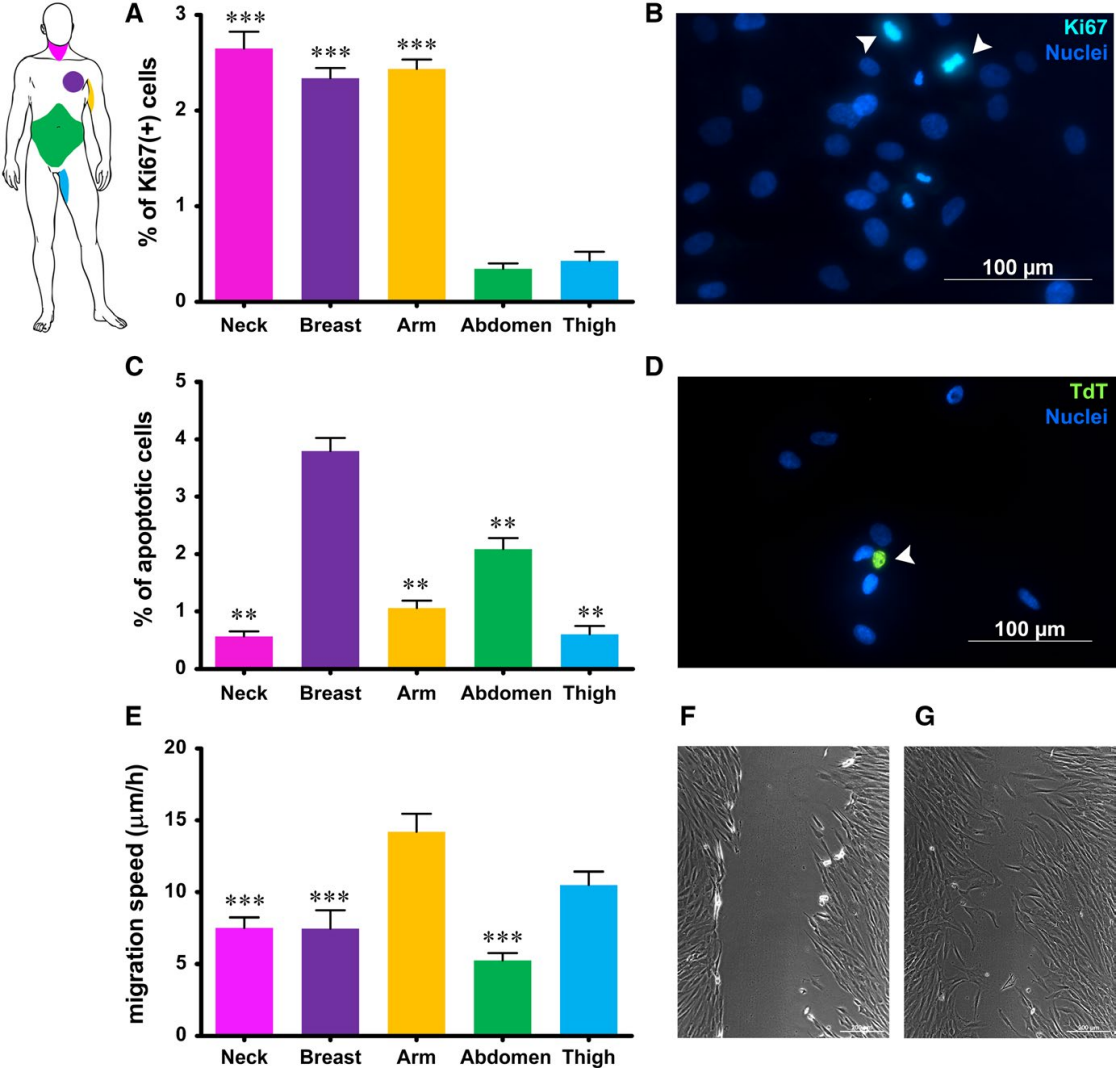
Quantification of proliferation index (A), apoptotic rate (C) and speed of migration (E) of fibroblasts from different anatomic sites, measured by expression of Ki67, TUNEL assay and scratch wound assay respectively. B, representative image of ki‐67‐positive cells in fibroblast culture (arrowheads); D: representative image of an apoptotic cell in fibroblast culture (arrowhead); (F) and (G) representative images of scratch wound assay of fibroblasts in culture at time 0 and after 12 h. Each value in graphs expresses the mean + SE of fibroblasts obtained from five different patients for each region (n = 5). Asterisks are indicators of the *P* value as follows: (A) extremely significant (***P ≤ 0.001) vs fibroblasts from abdomen and thigh; (C) **very significant (**P ≤ 0.01) vs fibroblasts from breast; (E) ***extremely significant (***P ≤ 0.001) vs fibroblasts from arm. (B) and (D) Scale bar length is 100 µm; (F) and (G) Scale bar length is 200 µm. At the upper left corner a scheme of anatomic sites of origin of fibroblasts is reported for quick reference

To avoid any effect due to native environment, all primary fibroblasts were cultured under the same condition in vitro for five passages. Proliferation index, calculated as the percentage of cycling cells detected by the expression of ki67, was significantly lower (*P* ≤ 0.05) in fibroblasts from abdomen and thigh, than in fibroblasts from all other regions, being as low as 0.347 ± 0.094% in fibroblasts from abdomen, and as high as 2.646 ± 0.303% in fibroblasts isolated from the neck skin (Figure 2A,B). Similarly, apoptotic index, calculated as the percentage of apoptotic cells detected by TUNEL assay, varied among different fibroblast populations. In particular, rate of spontaneous apoptosis of dermal fibroblasts from both breast (3.792 ± 0.234%) and abdomen regions (2.089 ± 0.188%) was significantly higher (*P* ≤ 0.05) than the apoptotic rate of fibroblasts from the dermis of neck, arm and thigh regions (0.564 ± 0.090%, 1.059 ± 0,131% and 0.600 ± 0,148% respectively) (Figure 2 C,D). Moreover, fibroblasts from arm and thigh dermis migrated at higher speed (14.209 ± 3.769 and 10.499 ± 2.973 μm/h respectively) than fibroblasts from all other regions, whose speed of migration was comparable and ranging from 5.237 ± 1.749 μm/h, for fibroblasts from abdomen dermis, to 7.179 ± 2.449 μm/h, for fibroblasts from neck dermis (Figure 2E‐G).

### 3.3 Expression of mesenchymal and epithelial markers

**Figure 3:**
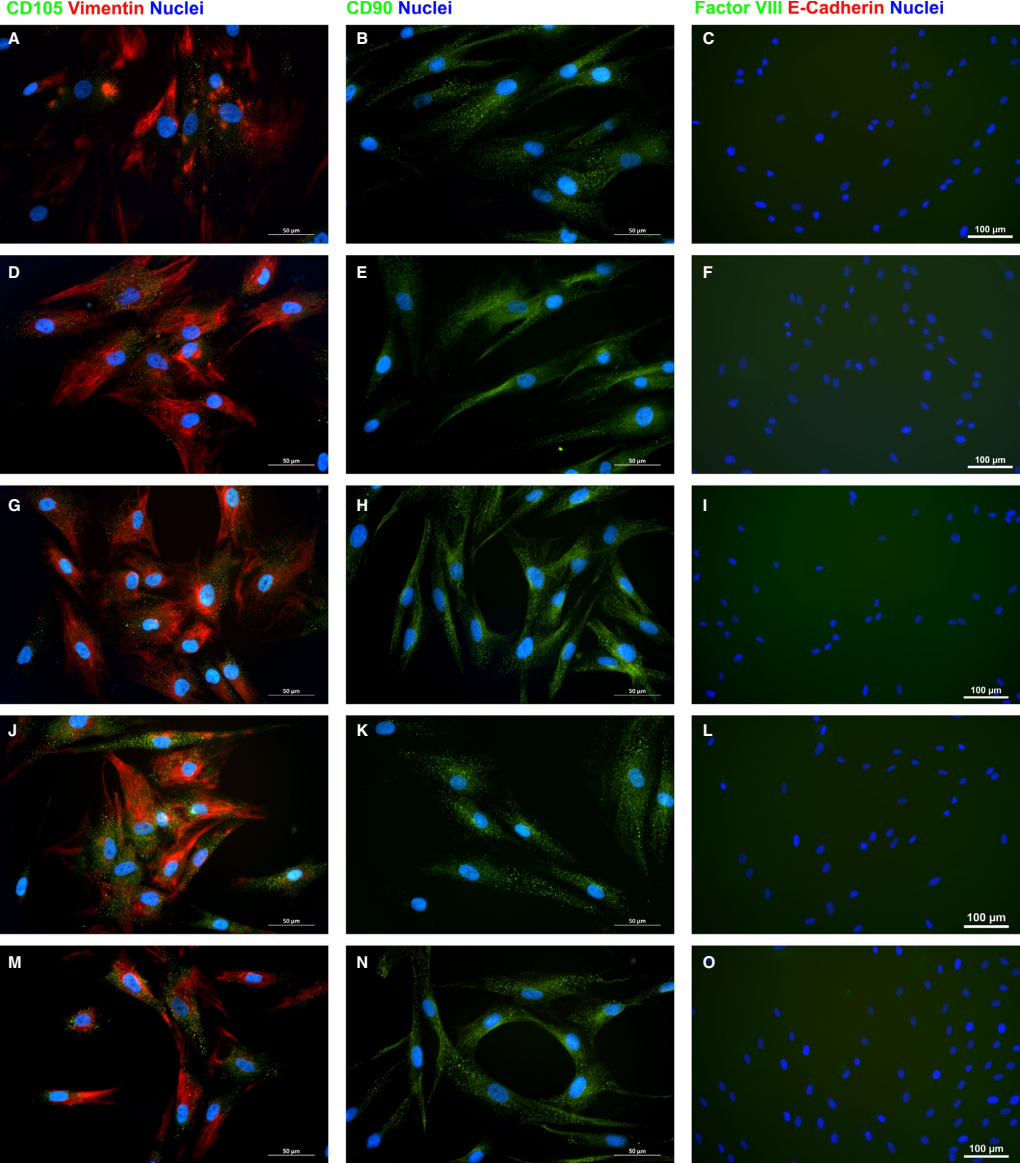
Representative images of immunocytochemistry for the in vitro expression of mesenchymal (A‐B, D‐E, G‐H, J‐K and M‐N) and epithelial (C, F, I, L and O) markers by dermal fibroblasts from neck (A‐C), breast (D‐F), arm (G‐I), abdomen (J‐L), and thigh (M‐O). Scale bar length is 50 (A‐B, D‐E, G‐H, J‐K and M‐N) or 100 µm (C, F, I, L and O)

**Figure 4:**
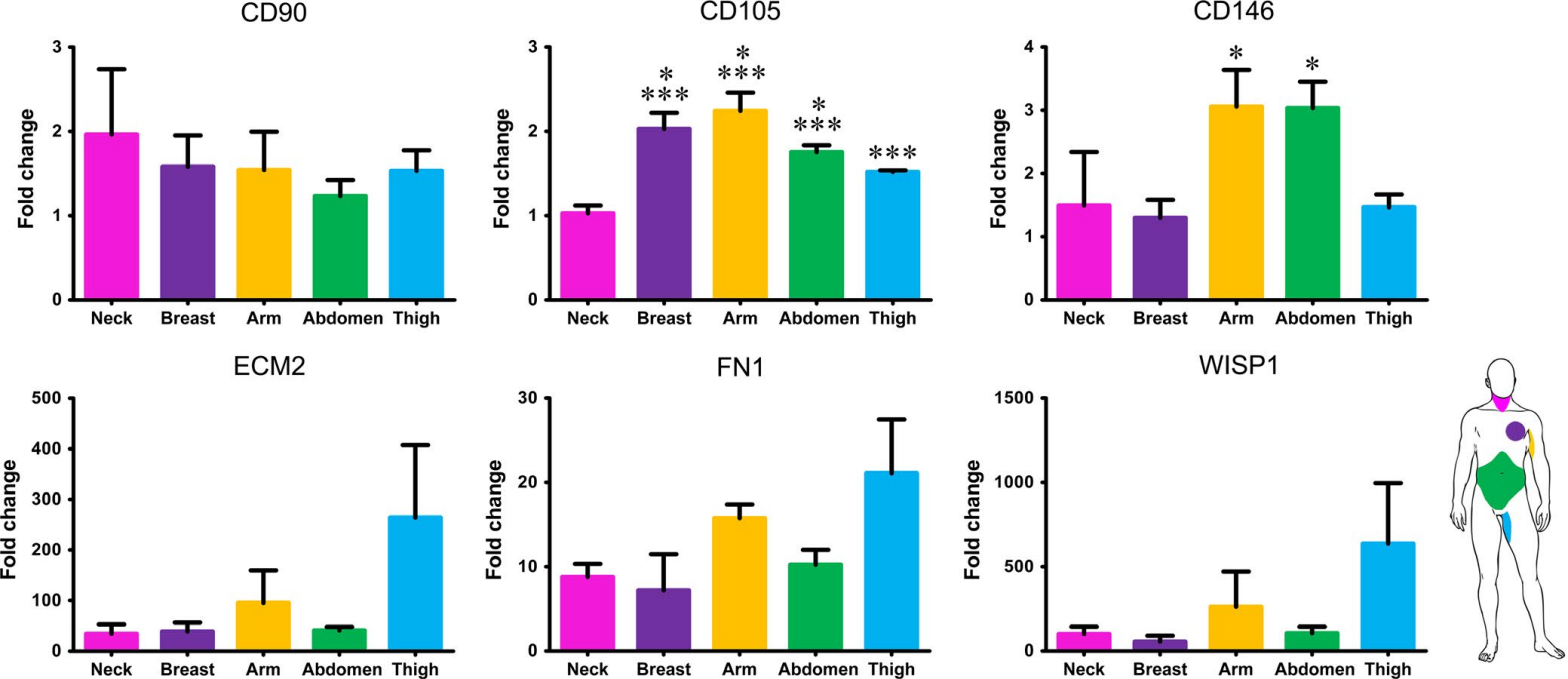
Real‐time PCR analysis of the expression of genes characteristic of mesenchymal cells. Each value expresses the mean of five samples for each region (n = 5) + SE. Asterisks are indicators of the *P* value as follows: CD105: significant (*P ≤ 0.05) vs fibroblasts from thigh and extremely significant (***P ≤ 0.001) vs fibroblasts from abdomen; significant (* *P* ≤ 0.05) vs fibroblasts from breast and thigh. WISP1, WNT1 inducible signaling pathway protein 1; FN1, Fibronectin 1; ECM2, Extracellular Matrix Protein 2.

All fibroblasts in culture were negative for epithelial and endothelial markers like E‐cadherin and Factor VIII (Figure 3). As for mesenchymal markers, variability related to anatomic site of origin occurred. Specifically, based upon vimentin and CD105 expression, fibroblasts in culture were either mainly vimentin‐positive or mainly CD105‐positive (Figure 3). Interestingly, the proportion of such subpopulations of fibroblasts varied according to the anatomic site: fibroblasts isolated from neck and breast resulted mostly vimentin‐positive (Figure 3 A,D), while fibroblasts from the skin of abdomen and thigh consisted of mostly CD105‐positive cells (Figure 3 J,M). No apparent difference among fibroblasts isolated from different regions emerged in the analysis of the expression of CD90, instead (Figure 3). Additionally, gene expression analysis by qRT‐PCR confirmed the presence of gene transcripts for mesenchymal markers. CD90, ECM2, FN1 and WISP1 were expressed at similar levels among dermal fibroblasts isolated from different anatomic sites. Statistically significant differences (*P* ≤ 0.05) were observed in the expression of CD105 and CD146, instead. Specifically, higher expression of CD105 was observed in fibroblasts from breast, arm and abdomen, while significantly higher expression of CD146 resulted in fibroblasts from arm and abdomen (Figure 4).

### 3.4 Mesenchymal differentiation and growth factor release

**Figure 5:**
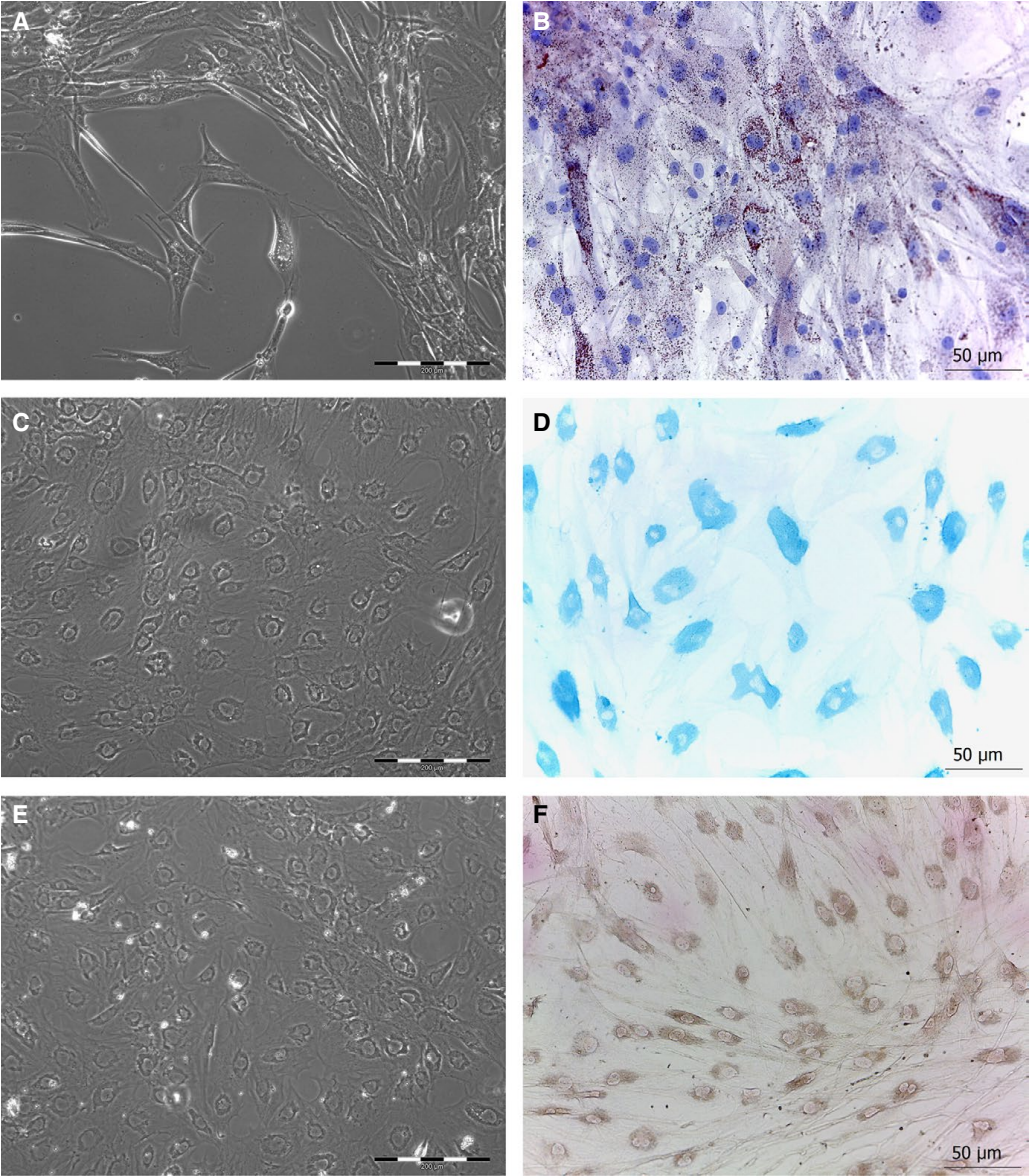
Following induction of adipogenic, chondrogenic and osteogenic differentiation, fibroblasts exhibited a dramatic change of phenotype as shown by representative images at phase‐contrast microscope (A, C, E). Specific stainings to identify intracellular lipid accumulation (Oil red O), sulfated glucose‐aminoglycan (Alcian blue) or calcium deposits (Von Kossa) confirmed the differentiation towards adipocytes (B), chondrocytes (D) and osteoblasts (F) respectively. Scale bar length is 200 µm for phase‐contrast images and 50 µm for brightfield images

**Figure 6:**
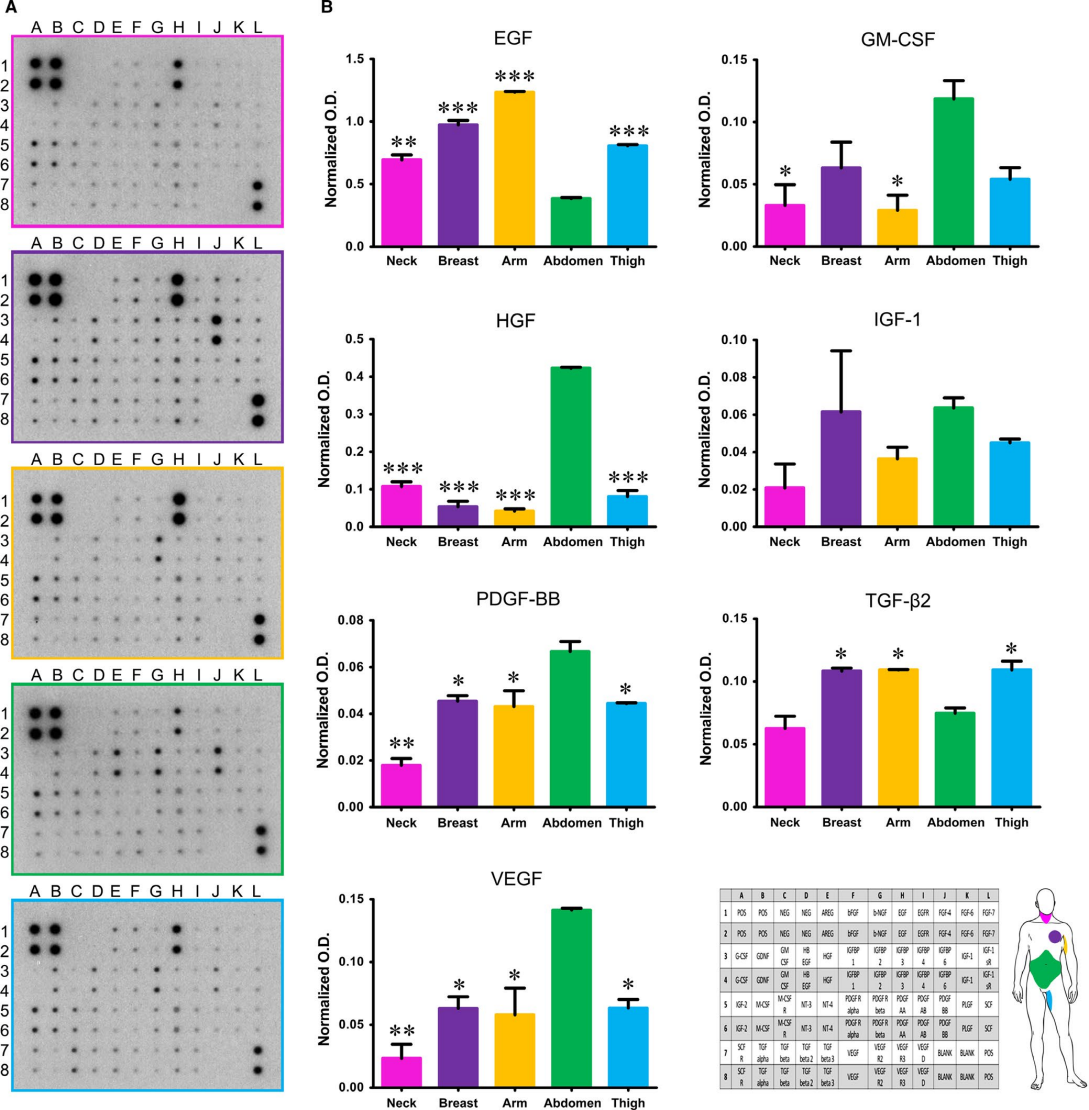
Production and release of growth factors by dermal fibroblasts in vitro evaluated by growth factor protein array. A, Representative images of profiles of growth factors released by fibroblasts from different anatomic sites. B. Quantification of selected growth factors. Each value expresses the mean + SE. Culture medium from culture of fibroblasts obtained from three different patients for each site were assayed (n = 3). Asterisks are indicators of the *P* value as follows: significant (*P ≤ 0.05), very significant (**P ≤ 0.01), and extremely significant (***P ≤ 0.001) vs fibroblasts from abdomen (EGF, GM‐CSF, HGF, PDGF‐BB and VEGF) or fibroblasts from neck and abdomen (TGF‐β2). At the lower right corner layout of protein array and a scheme of anatomic sites of origin of fibroblasts are reported for quick reference. EGF, Epidermal Growth Factor; OD, optical density; HGF, Hepatocyte Growth Factor; VEGF, Vascular Endothelial Growth Factor; GM‐CSF, Granulocyte‐macrophage colony‐stimulating factor; PDGF‐BB, Platelet‐Derived Growth Factor‐BB

To test the ability to differentiate, human dermal fibroblasts were grown in culture medium supplemented with horse serum or with Transforming Growth Factor (TGF‐β) or with dexamethasone, ascorbic acid and β‐glycerophosphate. Even though fibroblasts isolated from all different regions clearly retained the ability to differentiate towards chondrocytes, osteoblasts and adipocytes, as shown by specific histochemical stainings (Figure 5), obvious change of phenotype in culture in fibroblasts isolated from the skin of abdominal region was apparent as early as 6 days of culture, while fibroblasts from all other regions required a time ranging from 10 to 15 days. The presence of specific growth factors capable of enhancing or impairing reprogramming was detected by protein array performed on culture medium obtained from fibroblasts isolated from different anatomic sites. Statistically significant difference was observed in the release (*P* ≤ 0.05) of growth factors like Epidermal Growth Factor (EGF), Hepatocyte Growth Factor (HGF), Platelet‐Derived Growth Factor (PDGF), TGF‐β, Vascular Endothelial Growth Factor (VEGF) among fibroblasts of different origin (Figure 6). Specifically, dermal fibroblasts from abdominal skin released higher amounts of HGF, PDGF and VEGF, but significantly lower amounts of EGF and TGF‐β.

### 3.5 Reprogramming of dermal fibroblasts

**Figure 7:**
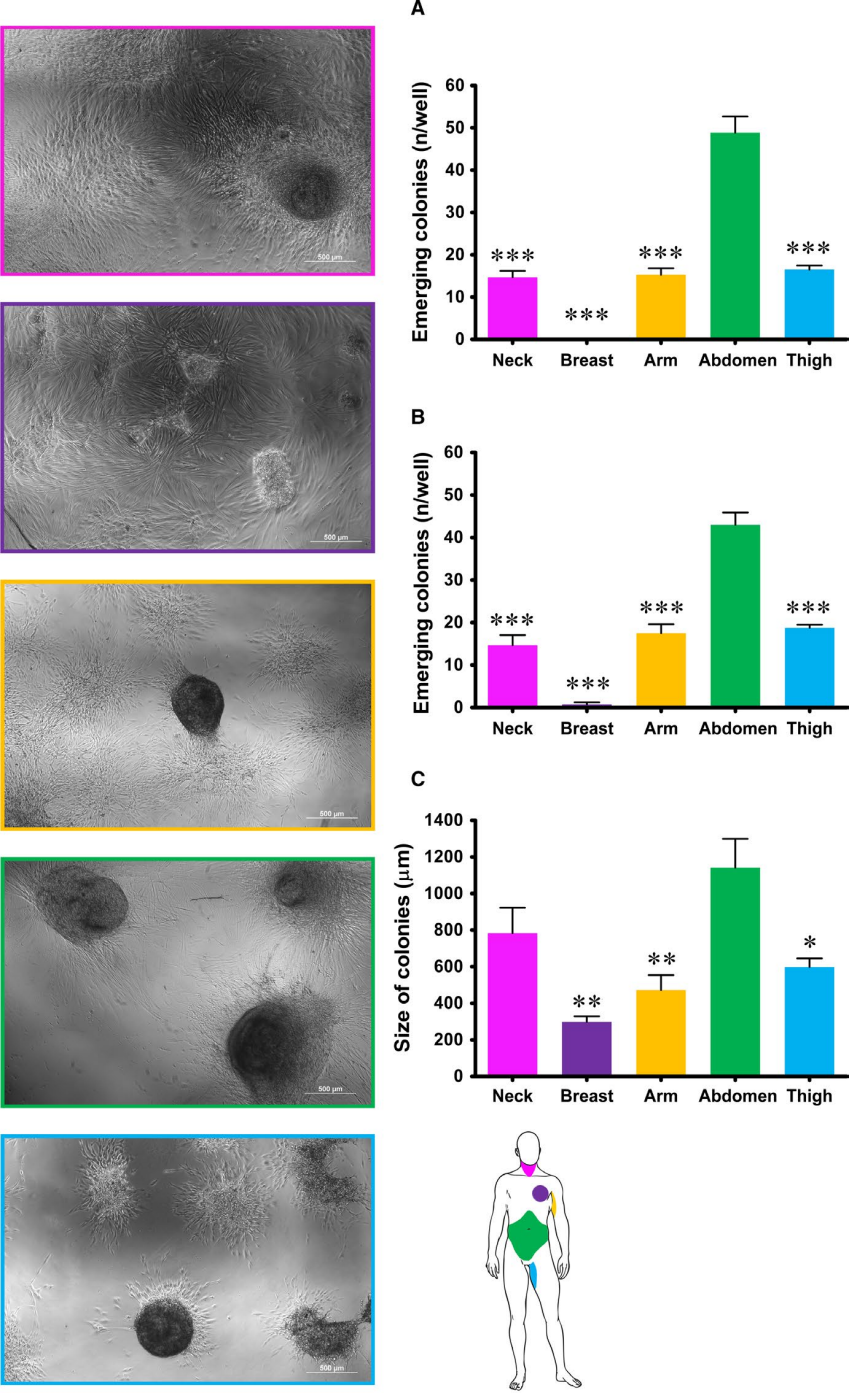
Reprogrammed human dermal fibroblast by mRNA direct delivery. On the left: representative images of colonies of induced pluripotent stem cells formed by fibroblasts from all five different anatomic sites at day 14 after transfection. On the right: (A) and (B) quantification of colonies formed by fibroblasts from the five different anatomic regions at day 7 (A) and 14 (B) after transfection. Each value expresses the mean ± SE (n = 4) and the three asterisks are indicators of the *P* value as extremely significant (*** *P* ≤ 0.001) vs fibroblasts from the abdominal skin. C, measurement of the size of colonies, expressed as the maximum diameter in μm, formed by fibroblasts from the five different anatomic regions at day 14 after transfection. Each value expresses the mean ± SE (n = 4) and asterisks are indicators of the *P* value as follows: significant (*P ≤ 0.05), very significant (**P ≤ 0.01) vs fibroblasts from the abdominal skin. At the bottom of the right side of the picture a scheme of anatomic sites of origin of fibroblasts is reported for quick reference

As a proof of concept we reprogrammed dermal fibroblasts using a ready‐to‐use kit and following the well‐established protocol supplied with the reagents.^25^, ^26^, ^27^ The efficiency of reprogramming, in terms of time required for the formation of colonies and of number and size of colonies, varied enormously among fibroblasts and in accordance with the anatomic site of origin (Figure 7). Fibroblasts isolated from the neck, arm and thigh regions required 1 week after the last transfection for reprogramming, while fibroblasts isolated from the breast skin reprogrammed after 2 weeks and dermal fibroblasts from the abdomen region started arranging into small colonies after the very first transfection. As regards the number of colonies that formed 7 and 14 days after transfection, no statistically significant differences emerged between fibroblasts isolated from the neck, arm or thigh skin after 7 days (14.17 ± 1.515, 15.29 ± 1.523 and 16.50 ± 0.964 respectively) nor after 14 days (14.67 ± 2.404, 17.50 ± 2.102 and 18.75 ± 0.75 respectively). Fibroblasts isolated from the breast skin formed colonies only after 14 days and their number was significantly lower (0.75 ± 0.491), while fibroblasts isolated from the abdominal skin formed a significantly higher number of colonies than fibroblasts from all other regions, both after 7 and 14 days (48.58 ± 3.843 and 43.00 ± 2.887 respectively). Finally, the size of colonies at day 14 after transfection was also measured and the mean maximum diameter of colonies formed varied greatly, ranging from about 0.3 mm to over 1 mm. Specifically, fibroblasts isolated from the abdominal skin formed the largest colonies (1141 ± 158.4 μm) that also resulted significantly larger than those formed by fibroblasts from breast (297.5 ± 31.26 μm), arm (472.0 ± 81.81 μm) and thigh (598.4 ± 46.28 μm) skin, while no statistically significant difference was observed in the size of colonies formed by fibroblasts from the skin of abdomen or neck (783.3 ± 139.5 μm).

## 4 DISCUSSION

Induced pluripotent stem cells are clearly emerging as the most promising cell type in regenerative medicine, as they are autologous cells characterised by pluripotency comparable to that of ESCs. Nonetheless, several hurdles related to reproducibility and efficiency of reprogramming are yet to be overcome. Fibroblast was the first adult somatic cell to be successfully reprogrammed and thus far it is still the most used cell for reprogramming. Noteworthily, it has been previously demonstrated that fibroblast are not a homogenous population^28^ and that cell origin and characteristics affect cell reprogramming.^17^ However, nor fibroblast features were evaluated nor the site of origin of fibroblasts was accurately selected before proceeding with the reprogramming. The present study aims at comparing and analysing features of human dermal fibroblasts that may impact efficiency of reprogramming, to point out how important it is to accurately select the fibroblast population to improve reprogramming efficiency.

Dermal fibroblasts from five different anatomic sites (neck, breast, arm, abdomen and thigh) were isolated and cultured under the same culture conditions. All fibroblasts adhered and grew on plastic culture dishes, lacked expression of markers specific of other cell lineages and, in accordance with previous report of different morphology in vitro,^28^ their morphology varied from elongated and spindle‐shaped to flattened and star‐shaped (Figure 1). Nevertheless, several other features, such as the expression of mesenchymal markers, speed of migration, proliferation index, promptness to acquire different phenotype and release of growth factors, varied greatly among dermal fibroblasts isolated from different anatomic sites. Based on expression of mesenchymal markers, fibroblasts in culture consisted of either mostly vimentin‐positive or mostly CD105‐positive cells (Figure 3 and Figure 4). Such evidence was in agreement with fibroblast diverse ability to migrate, as vimentin is the mesenchymal intermediate filament essential to ensure migration.^20^, ^29^ Indeed, mostly vimentin‐positive fibroblasts isolated from the arm skin migrated at considerable higher speed than all other fibroblast populations (Figure 2). Additionally, consistently with previous report of vimentin‐deficient fibroblasts exhibiting a slower rate of proliferation,^21^ fibroblasts isolated from the arm skin, along with fibroblasts from neck and breast regions, all mostly vimentin‐positive, proliferated at significantly higher rate than dermal fibroblasts from abdomen or thigh that showed a reduced expression of vimentin (Figure 2). Notably, accumulating evidence suggests that low proliferation rates of somatic cells enhance their reprogramming,^30^, ^31^, ^32^ hence difference in proliferation rate of fibroblasts isolated from different anatomic sites might indirectly suggest a diverse response of fibroblast populations to reprogramming. Despite the common expression of several transcripts of genes typically expressed in mesenchymal cells,^33^, ^34^ there was a heterogeneous expression of CD105 and CD146 when comparing dermal fibroblasts obtained from distinct anatomic sites, with fibroblasts isolated from abdomen skin that had a higher expression of all three markers. Our data are in disagreement with the previous proposal of using CD146 as a marker of mesenchymal stromal/stem cells (MSCs) that is based on the evidence that fibroblasts are CD146‐negative.^34^, ^35^ However, heterogeneity of MSCs as well as evidence that MSCs and fibroblasts cannot be unequivocally distinguished in vitro have also been described.^36^ Indeed, apart from sharing most of the features and expression of markers of fibroblasts, MSCs, like fibroblasts, exhibit striking variability among tissues of origin and donors that make even more difficult to define a subset of markers to clearly identify them,^37^, ^38^, ^39^ to such an extent that MSCs isolated from adipose tissue have been described as a CD146‐negative cell population.^40^, ^41^ Interestingly though, expression of CD90, CD105 and CD146 by fibroblasts isolated from abdominal skin raise the thorny, and still unaddressed, question of whether fibroblasts and MSCs are not admittedly the same population.^42^, ^43^ Based on our and other authors' observations,^33^, ^34^, ^41^ it is reasonable to consider fibroblasts to be at least an MSC differentiation stage, and to infer that fibroblast cell population comprises a variable number of MSCs, whose proportion is related to the anatomic site of origin and could cause the dermal fibroblasts from different anatomic sites to respond to reprogramming technology in different manner. This hypothesis is fully supported and strengthened by conflicting evidence of fibroblast ability to differentiate towards chondrocytes, osteoblasts and adipocytes.^41^, ^44^, ^45^, ^46^ Although retained ability of dermal fibroblasts to differentiate into chondrocytes, osteoblasts and adipocytes emerged from our analysis (Figure 5), obvious difference emerged among fibroblasts from different regions. Indeed, change of phenotype in fibroblasts isolated from the skin of abdominal region was apparent as early as 6 days of culture, while fibroblasts from all other regions required a time ranging from 10 to 15 days, confirming that the site of origin is responsible for functional diversity of dermal fibroblast and that it is, realistically, related to a diverse proportion of MSCs in fibroblast cell culture, as MSCs are unquestionably capable of giving rise to all three mesenchymal cell lines. Interestingly, the presence of specific growth factors capable of enhancing or impairing reprogramming, 47, 48, 49, 50 as detected by protein array performed on fibroblasts isolated from different anatomic sites, provided additional clue about diversity of fibroblasts and their diverse ability to respond to reprogramming technology. Notably, the release of the EGF, proved to be an enhancer of fibroblast proliferation^51^ and, as consequence, of impairing cell reprogramming,^30^ was significantly lower in culture of slow‐proliferating dermal fibroblast from abdominal skin and significantly higher in culture of fast‐proliferating fibroblasts from arm skin. On the contrary, the release of growth factors known to be inducers of cell reprogramming, like GM‐CSF,^52^ HGF,^53^ PDGF 54, 55 and VEGF 49 resulted significantly higher in dermal fibroblasts from abdominal skin (Figure 6). Furthermore, as reprogramming of fibroblasts to iPSCs requires a mesenchymal to epithelial transition (MET),^56^ factors involved in the activation of MET or inhibition of the opposite process, the epithelial‐to‐mesenchymal transition (EMT), have potential critical role in reprogramming. TGF‐β is a known inducer of EMT and its inhibition has been correlated with the enhancement of MET and reprogramming.^50^ Intriguingly, the release of TGF‐β by fibroblasts from abdomen skin was significantly lower.

Evidence discussed thus far substantiates the hypothesis that human dermal fibroblasts derived from different sites have different features, including expression of mesenchymal markers, proliferation index, promptness to differentiate among mesenchymal cell lineages and production and release of growth factor. As all these features might remarkably affect the efficiency of reprogramming, we inferred that fibroblast topographic location might impact upon induction and efficiency of reprogramming. Hence, as a proof of concept we induced the reprogramming of dermal fibroblasts from all five anatomic sites following a well‐established mRNA‐based protocol. Remarkable differences in efficiency of reprogramming, in terms of ability to form colonies, emerged from reprogramming experiments. Indeed, fibroblasts isolated from the skin of the abdominal region started reprogramming as early as day 2, while fibroblasts from other regions took between 11 and 18 days to form colonies. Moreover, the number and size of colonies formed were dramatically and significantly higher in fibroblasts from the abdominal skin (Figure 7) that in fibroblasts from all other regions, providing evidence of higher efficiency of reprogramming. Therefore, fibroblast reprogramming is more readily and efficiently accomplished from abdominal skin fibroblasts that are facilely and efficiently harvested by a skin punch biopsy that may be performed with a minor unaesthetic scar.

In conclusion, while offering novel perspective on the iPSC reprogramming from dermal fibroblasts, our study also demonstrates that fibroblasts isolated from the skin of the abdomen represent the ideal dermal fibroblast for. As a result of their ESC‐like pluripotency, iPSCs are currently considered the most promising cell source for regenerative medicine applications. Nonetheless, severe technical problems related to both reprogramming technology and low efficiency of reprogramming still need to be addressed before clinical translation. While the integration‐free methods like the reprogramming induced by direct delivery of modified mRNA employed in this study, with the possibility of applying the protocol in a complete xeno‐free culture environment, ensures safety for iPSC translation into clinical settings, the identification of a dermal fibroblast population that promptly responds to reprogramming procedure by quickly forming a number of large and well‐structured colonies has the tremendous potential of advancing iPSC technology and boost their use in clinical trials.

Additionally, while emphasizing fibroblast diversity and the impact of such diversity on cell reprogramming, our study also highlights striking similarity between fibroblasts and MSCs that supports the attracting hypothesis that the most remarkable difference between fibroblasts and MSCs is in the name.^40^, ^41^, ^42^, ^43^, ^57^

## CONFLICT OF INTEREST

The authors have no conflicts of interest to disclose.

## AUTHOR CONTRIBUTION

AMS and IB contributed equally to this work. FDM and CC are joint senior authors.
